# Establish a Nomogram to Predict Falls in Spinocerebellar Ataxia Type 3

**DOI:** 10.3389/fneur.2020.602003

**Published:** 2021-01-27

**Authors:** Junyu Lin, Lingyu Zhang, Bei Cao, Qianqian Wei, Ruwei Ou, Yanbing Hou, Xinran Xu, Kuncheng Liu, Xiaojing Gu, Huifang Shang

**Affiliations:** Department of Neurology, Laboratory of Neurodegenerative Disorders, Rare Diseases Center, West China Hospital, Sichuan University, Chengdu, China

**Keywords:** spinocerebellar ataxia type 3, eye movements, falls, nomogram, prospective

## Abstract

**Purpose:** Falls are common and are frequently accompanied by injuries in patients with spinocerebellar ataxias type 3 (SCA3). We explored which factors could predict falls in a cohort of patients with SCA3 and developed a nomogram model to predict the first fall in non-fallen patients with SCA3.

**Method:** We conducted a prospective cohort study. Forty-four non-fallen patients with SCA3 were followed up until the first fall or November 5, 2020, whichever came first. Univariate and multivariate Cox proportional hazard regression analyses were applied to explore the predictive factors of falls in patients with SCA3. A nomogram model predicting the no-fall probabilities at 3, 6, 12, and 24 months was formulated based on the results of the multivariate Cox analysis. Internal validation was conducted to assess the discrimination and calibration of the final model using bootstrapping with 1,000 resamples.

**Results:** Multivariate Cox proportional hazard regression showed that the presence of dystonia, hyperreflexia, urinary incontinence, and hidrosis and the number of abnormal eye movements predicted a more rapid progression to falls in patients with SCA3. The nomogram model showed good discrimination with a concordance index of 0.83 and good calibration.

**Conclusion:** Patients with dystonia, hyperreflexia, urinary incontinence, and hidrosis, and more types of abnormal eye movement had a more rapid progression to falls in SCA3.

## Introduction

Spinocerebellar ataxias (SCAs) are a genetically heterogeneous group of autosomal dominantly inherited progressive disorders characterized neuropathologically by degeneration of the cerebellum and clinically by loss of balance and coordination accompanied by slurred speech. Spinocerebellar ataxias type 3 (SCA3), also known as Machado-Joseph disease, is the most common SCA worldwide and is caused by CAG expansion of the *ATXN3* gene. In China, SCA3 is also the most common subtype of SCA, accounting for 48–49% of patients with SCA (1).

Falls are frequent in patients with SCA (2). In a study including 42 patients with autosomal dominant cerebellar ataxias or idiopathic late-onset cerebellar ataxias, up to 93% of patients reported at least one fall in the 12 preceding months. Another study observed that 84.1% of patients experienced at least one fall during a period of 12 months in a cohort of patients with SCA1, SCA2, SCA3, or SCA6 with a mean disease duration of 11.8 ± 6.4 years (3). In addition, falls are frequently accompanied by serious injuries. Eighty-five percent of the fallers reported injuries, and 31% of the fallers suffered a fracture or joint dislocation among patients with degenerative cerebellar ataxias (4). Falls may also induce a fear of falling and can cause a vicious cycle of immobilization.

According to a previous study, falls can occur as early as 2 years after disease onset in SCA (4). A previous retrospective study explored the factors associated with falls in patients with SCA1, SCA2, SCA3, and SCA6 and determined that disease duration, severity of ataxia, presence of pyramidal symptoms, total number of non-ataxia symptoms, and genotype SCA3 were associated with a higher fall frequency (2). However, no prospective study has been conducted as yet to explore the risk factors that could predict the first fall in non-fallen patients with SCA3. Nomograms, visible, and effective tools for multivariate survival analysis, have been widely used in cancer and other diseases (5). To date, no nomogram has been applied to predict falls in patients with SCA3. Therefore, in the current study, we aimed to investigate which factors could predict falls in a cohort of patients with SCA3 and to develop a nomogram model to predict the first fall in non-fallen patients with SCA3.

## Materials and Methods

### Patients Evaluation

This study was executed in agreement with the Ethics Committee of West China Hospital of Sichuan University. All recruited participants provided written informed consent. A total of forty-four genetically confirmed non-fallen patients with SCA3 (16 males) were consecutively recruited from the Department of Neurology, West China Hospital of Sichuan University, between August 2015 and January 2020.

We performed a prospective study in the current study. All patients received a genetically confirmed diagnosis of SCA3 after testing for trinucleotide repeat expansions of genes causing *SCA1, SCA2, SCA3, SCA6*, and *SCA7* using short tandem repeat (STR) analysis. The CAG repeat lengths of the expanded allele were collected. All patients underwent a face-to-face interview at baseline. Sex, age, weight, height, educational years, age at onset, and disease duration of these patients were collected. The body mass index (BMI) was calculated by body weight (kg) divided by height squared (m^2^). The Montreal Cognitive Assessment (MoCA) was used to assess global cognitive function (6). Depression was screened using the Hamilton Depression Rating Scale-24 (HDRS-24) (7). Anxiety was screened using the Hamilton Anxiety Rating Scale (HARS) (8). Excessive daytime sleepiness was screened using the Epworth Sleepiness Scale (ESS) (9). Sleep problems were evaluated using the Pittsburgh sleep quality index (PSQI) (10).

Ataxia severity was assessed using the Scale for the Assessment and Rating of Ataxia (SARA), which is composed of eight items—gait, stance, sitting, speech disturbance, finger chase, nose-finger test, fast alternating hand movements, and heel-shin slide—and has been proven to be a reliable and valid clinical scale measuring the severity of ataxia (11). Non-ataxia symptoms, including bradykinesia, spasticity, paresis, muscle atrophy, fasciculation, myoclonus, rigidity, chorea/dyskinesia, dystonia, resting tremor, action tremor, sensory symptoms, dysphagia, hyperreflexia, urinary incontinence, constipation, hidrosis, orthostatic hypotension, diplopia, vision loss, and eye movement abnormalities, were evaluated by neurologists who were experienced in movement disorders. Eye movement abnormalities, including impaired smooth pursuit, increased square-wave jerks (SWJ), gaze-evoked nystagmus (GEN), slowing of saccades, saccadic hypo/hypermetria, and supranuclear gaze palsy, were evaluated using accepted bedside techniques (12). Impaired smooth pursuit, GEN, slowing of saccades, and saccadic hypo/hypermetria were evaluated following the International Cooperative Ataxia Rating Scale (ICARS) procedure (13). SWJ was detected during central fixation, and increased SWJ was defined as SWJ ≥ 10 per minute (14). Pursuit, saccade speed, saccade accuracy, supranuclear gaze palsy, and GEN were evaluated in both the horizontal and vertical planes.,

The 44 patients with SCA3 were followed up with telephone or face-to-face interviews with an irregular interval. November 5, 2020, was the last follow-up time. The endpoint was set as the first fall or November 5, 2020, whichever came first.

### Statistical Analysis

Since no continuous variables were normally distributed, they were presented as the mediums (ranges), and all categorical variables were presented as numbers and percentages. Survival analysis was conducted to explore which factors could predict falls as early biomarkers. The fall-free time from enrollment was calculated from the date of enrollment to the first fall for fallers and from the date of enrollment to the date of last follow-up, namely, November 5, 2020, for non-fallers. We used the time from enrollment as the timescale. Thirty-two variables were evaluated for their predictive role for falls using univariate Cox proportional hazard regression models, including age, sex, educational year, disease duration, CAG repeat numbers, BMI, SARA score, MoCA score, HARS score, HDRS-24 score, PSQI score, ESS score, the number of abnormal eye movement, vision loss, bradykinesia, spasticity, paresis, muscle atrophy, fasciculation, rigidity, chorea/dyskinesia, dystonia, resting tremor, action tremor, sensory symptoms, dysphagia, diplopia, orthostatic hypotension, hyperreflexia, urinary incontinence, constipation, and hidrosis. The p-values were false discovery rate (FDR)-corrected for multiple testing to avoid false positive significances. Then, candidates with a FDR-corrected *p* < 0.20 were entered into the multivariate Cox proportional hazard regression model. In addition, given that age, disease duration, CAG repeat numbers, and abnormal eye movement have been reported to be prognostic factors for the survival of SCA3 patients (15), they were always included in the multivariate Cox proportional hazard regression model regardless of the p value obtained in univariate analysis. Finally, a nomogram model predicting the no-fall probabilities at 3, 6, 12, and 24 months was formulated based on the results of the multivariate Cox analysis. Internal validation was conducted to assess the discrimination and calibration of the nomogram model using bootstrapping with 1,000 resamples. Discrimination was measured with the concordance index (c-index) and the time-dependent receiver operating characteristic curve (t-ROC); the larger the c-index or the areas under the ROC curve (AUC) was, the more accurate the prognostic prediction was. Calibration curves were plotted to assess the calibration of the nomogram. All analyses were performed using the Statistical Package for the Social Sciences (SPSS) version 22.0 and R version 4.0.2. Two-tailed p values of <0.05 were considered statistically significant.

## Results

**Figure 1:**
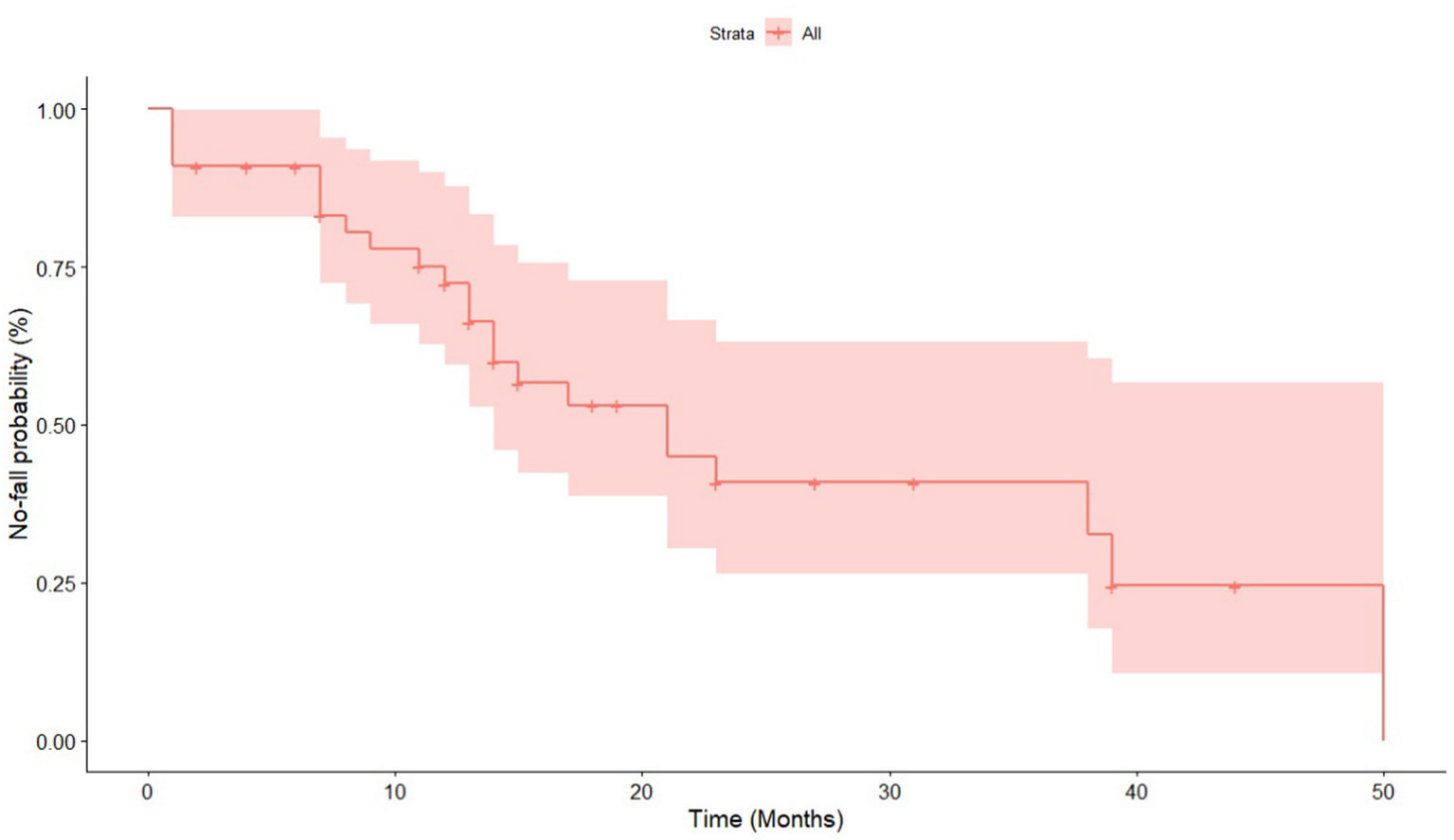
Kaplan-Meier graph of the first fall in patients with SCA3.

**Figure 2:**
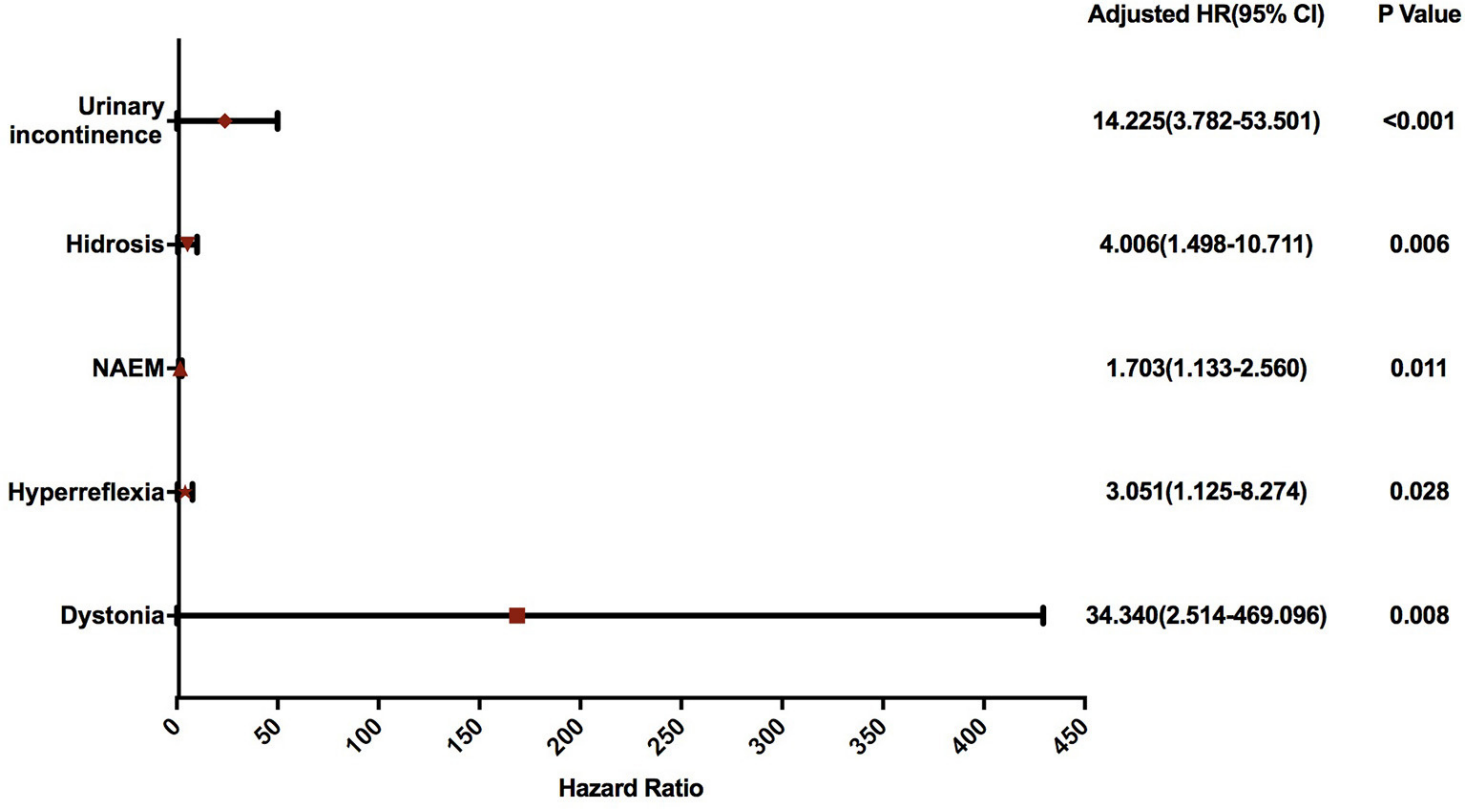
Forest plot of the hazard ratio for fall indicators analyzed using a multivariate Cox proportional hazard regression model. SCA3, spinocerebellar ataxia 3; HR, hazard ratio; NAEM, number of abnormal eye movements.

All forty-four patients were followed up until the first fall or November 5, 2020, whichever came first (Table 1). The mean follow-up time was 16.23 ± 12.35 months. The Kaplan-Meier graph is shown in Figure 1. In the univariate Cox proportional hazard regression models applied, four variables were identified to have a FDR-corrected *p* < 0.20, including dystonia (*p* = 0.168), hyperreflexia (*p* = 0.168), urinary incontinence (*p* < 0.032), and hidrosis (*p* = 0.112) (Table 2). Combined with age, disease duration, CAG repeat numbers, SARA score, and the number of abnormal eye movement, nine variables were ultimately included in the multivariate Cox proportional hazard regression model. The multivariate Cox proportional hazard regression showed that the number of abnormal eye movements (HR = 1.703, 95% CI: 1.133–2.560, *p* = 0.011), dystonia (HR = 34.340, 95% CI: 2.514–469.096, *p* = 0.008), hyperreflexia (HR = 3.051, 95% CI: 1.125–8.274, *p* = 0.028), urinary incontinence (HR = 14.225, 95% CI: 3.782–53.501, *p* < 0.001), and hidrosis (HR = 4.006, 95% CI: 1.498–10.711, *p* = 0.006) predicted a more rapid progression to falls in SCA3 patients (Table 3 and Figure 2).

**Table 1:** Demographic and clinical features of the recruited patients with SCA3.

**Variable**	**Prospective cohort study**
	(*n* = 44)
Sex (male,%)	16 (36.4%)
Mean age (years)	46.0 (23.0–68.0)
Age at onset (years)	40.0 (22.0–66.0)
Disease duration (years)	3.0 (0.5–20.0)
Educational year	9.0 (0.0–20.0)
BMI	21.5 (16.4–33.8)
Diplopia (%)	16 (36.4%)
Vision loss (%)	15 (34.1%)
Rigidity (%)	27 (61.4%)
Bradykinesia (%)	13 (29.5%)
Resting tremor (%)	3 (6.8%)
Action tremor (%)	6 (13.6%)
Dystonia (%)	1 (2.3%)
Muscle atrophy (%)	6 (13.6%)
Chorea/dyskinesia (%)	1 (2.3%)
Sensory symptoms (%)	14 (31.8%)
Paresis (%)	14 (31.8%)
Spasticity (%)	10 (22.7%)
Fasciculation (%)	9 (20.5%)
Hyperreflexia (%)	20 (45.5%)
Dysphagia (%)	6 (12.2%)
Urine incontinence	6 (13.6%)
Constipation	4 (9.1%)
Hidrosis	15 (34.1%)
Orthostatic hypotension	10 (20.4%)
SARA score	9.0 (0.5–27.0)
MoCA score	24.5 (12.0–29.0)
HDRS-24 score	6.5 (0.0–43.0)
HARS score	5.0 (0.0–28.0)
ESS score	4.0 (0.0–15.0)
PSQI score	7.0 (1.0–19.0)
CAG repeat length	70 (59–77)

*SCA3, spinocerebellar ataxia type 3; BMI, body mass index; SARA, Scale for the Assessment and Rating of Ataxia; MoCA, Montreal Cognitive Assessment; HDRS-24, Hamilton Depression Scale; HARS, Hamilton Anxiety Scale; ESS, Epworth Sleepiness Scale; PSQI, Pittsburgh sleep quality index*.

**Table 2:** Univariate Cox proportional hazard regression analysis for falls in patients with SCA3.

**Variable**	**Univariate analysis**	
	**HR(95% CI)**	**FDR-correted *P*-Value**
Age	0.987 (0.949–1.027)	0.797
Sex	0.626 (0.254–1.545)	0.661
Educational year	1.015 (0.931–1.106)	0.841
Disease duration	1.016 (0.895–1.152)	0.841
BMI	0.986 (0.861–1.130)	0.841
Rigidity	1.546 (0.628–3.802)	0.661
Bradykinesia	1.117 (0.435–2.869)	0.841
Resting tremor	1.403 (0.314–6.262)	0.830
Action tremor	0.689 (0.160–2.978)	0.830
Dystonia	14.333 (1.491–137.794)	0.168
Muscle atrophy	2.197 (0.800–6.035)	0.498
Chorea/dyskinesia	2.110 (0.276–16.118)	0.795
Sensory symptoms	0.823 (0.333–2.037)	0.830
Paresis	0.890 (0.359–2.206)	0.841
Spasticity	1.804 (0.701–4.641)	0.551
Fasciculation	0.465 (0.135–1.594)	0.551
Hyperreflexia	2.860 (1.205–6.786)	0.168
Dysphagia	2.963 (1.071–8.200)	0.230
SARA score	1.070 (0.994–1.151)	0.384
MoCA score	1.033 (0.925–1.153)	0.823
HDRS-24 score	1.015 (0.981–1.050)	0.720
HARS score	1.024 (0.975–1.075)	0.661
PSQI score	0.971 (0.886–1.063)	0.797
ESS score	1.058 (0.966–1.159)	0.551
CAG repeat number	1.102 (0.970–1.252)	0.498
Number of abnormal eye movements	1.278 (0.922–1.770)	0.498
Vision loss	1.551 (0.651–3.693)	0.611
Diplopia	1.794 (0.771–4.173)	0.551
Urine incontinence	6.229 (2.238–17.334)	<0.032*
Constipation	1.251 (0.366–4.279)	0.841
Hidrosis	3.229 (1.381–7.550)	0.112
Orthostatic hypotension	1.262 (0.479–3.325)	0.830

*SCA3, spinocerebellar ataxia type 3; BMI, body mass index; SARA, Scale for the Assessment and Rating of Ataxia; MoCA, Montreal Cognitive Assessment; HDRS-24, Hamilton Depression Scale; HARS, Hamilton Anxiety Scale; PSQI, Pittsburgh sleep quality index; ESS, Epworth Sleepiness Scale; HR, hazard ratio*.

* *Significant difference after false discovery rate (FDR) correction for multiple testing*.

**Table 3:** Multivariate cox proportional hazard regression analysis for falls in patients with SCA3.

**Variable**	**Multivariate analysis**	
	**HR(95% CI)**	***P***
Dystonia	34.340(2.514–469.096)	0.008*
Hyperreflexia	3.051(1.125–8.274)	0.028*
Number of abnormal eye movements	1.703(1.133–2.560)	0.011*
Hidrosis	4.006(1.498–10.711)	0.006*
Urine incontinence	14.225(3.782–53.501)	<0.001*

*SCA3, spinocerebellar ataxia type 3; HR, hazard ratio*.

* *Significant difference. P value was calculated by a multivariate Cox proportional hazard regression model, with age, disease duration, SARA score, CAG repeat number, number of abnormal eye movement, dystonia, hyperreflexia, urine incontinence, and hidrosis were included as co-variables*.

**Figure 3:**
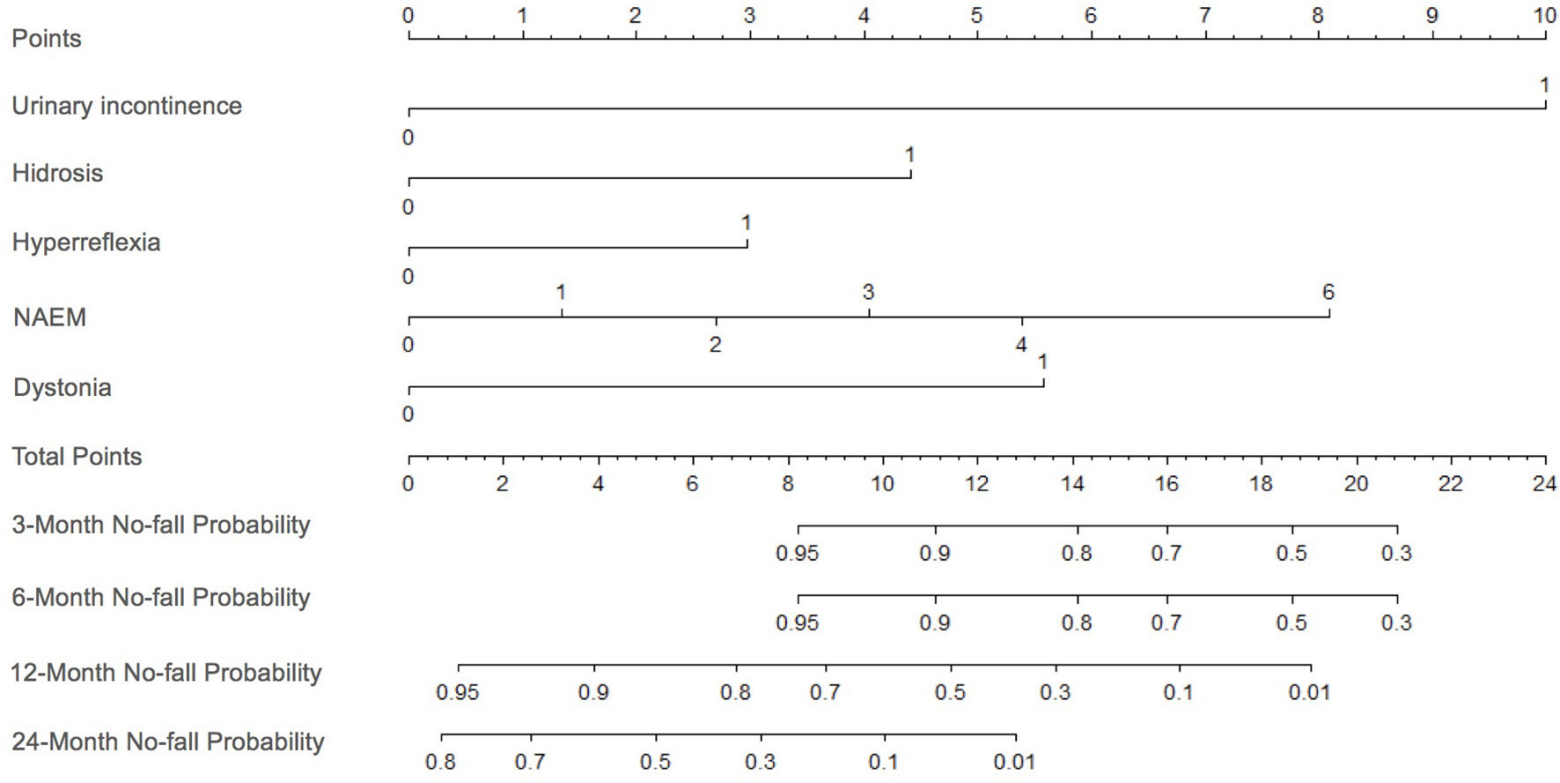
Nomogram plot to predict no-fall probability in patients with SCA3. The no-fall probability is calculated by drawing a line to the point on the axias for each of the following variables: dystonia, hyperreflexia, urinary incontinence, hidrosis, and the number of abnormal eye movements. The points for each variable are summed and located on the total points line. Then, a vertical line is projected from the total point line to the predicted probability bottom scales to obtain the individual no-fall probabilities. SCA3, spinocerebellar ataxia 3; NAEM, number of abnormal eye movements.

The presence of dystonia, hyperreflexia, urinary incontinence, and hidrosis and the number of abnormal eye movements were used to build the nomogram model according to the results of the multivariate Cox proportional hazard regression analysis (Figure 3). The c-index of the nomogram model was 0.83. In t-ROC analysis, the AUC for predicting 3, 6, 12, and 24 months no-fall probability were 0.919, 0.919, 0.971, and 0.841, respectively (Supplementary Figure 1). The calibration curves of the nomogram for the prediction of no-fall probability at 3, 6, 12, and 24 months showed good calibration (Supplementary Figure 2).

## Discussion

Fall frequency has been reported to be associated with disease duration, ataxia severity, presence of pyramidal symptoms, total number of non-ataxia symptoms (including hyperreflexia, areflexia, extensor plantar reflex, spasticity, paresis, muscle atrophy, fasciculation, myoclonus, rigidity, chorea/dyskinesia, dystonia, resting tremor, sensory symptoms, urinary dysfunction, cognitive dysfunction, and brainstem oculomotor signs), and the genotype of SCA3 in patients with SCA by correlation analysis (2, 3). The current study established the first nomogram model to predict falls in patients with SCA3. This model showed good discrimination and calibration. In this predictive model, dystonia, hyperreflexia, urinary incontinence, hidrosis, and abnormal eye movements were indicators for falls in patients with SCA3.

Dystonia is one of the most common extrapyramidal manifestations in SCA3. Previous studies have reported a prevalence of dystonia between 3.7 and 33.3% in SCA3 (16, 17). Dystonia and ataxia may share some overlapping pathologies, such as synaptic transmission and neurodevelopment (18). A study found that the presence of dystonia correlated with greater severity of ataxia in SCA3 (19). Another study revealed the predictive value of dystonia on survival in SCA3, which was consistent with our findings (20).

Hyperreflexia is present in 66.7%−78.3% of patients with SCA3 (21, 22). A previous study found that hyperreflexia was associated with longer CAG repeat lengths in SCA3 (23). Another study found that the pyramidal signs could predict a more rapid progression to death using the univariate Cox analysis (20). Our study confirmed the predictive role of hyperreflexia in disease progression.

Autonomic dysfunction is not rare in patients with SCA3 (24). A study reported a urinary incontinence prevalence of 39.3%, an orthostatic hypotension prevalence of 35.7%, and a hidrosis prevalence of 39.3% in patients with SCA3, which were significantly higher than those in healthy controls (25). A study revealed the involvement of Onuf's nucleus in SCA3 using morphometric and immunohistochemical methods, which may account for the autonomic dysfunction in SCA3 (26). However, the relationship between autonomic dysfunction and disease progression in SCA3 has never been studied before. Our study revealed the predictive role of autonomic dysfunction on falls in patients with SCA3 for the first time, emphasizing the importance of autonomic dysfunction screening when handling with patients with SCA3.

Abnormal eye movements are common in SCA3 (27). Several cross-sectional studies have shown the correlation between abnormal eye movements and disease severity in patients with SCA3 (28–30). The brain structures involved in the occurrence of abnormal eye movements are wide, and the neural network involved is complex (31). The flocculonodular lobe (32), the dorsal oculomotor vermis (OMV) (lobule VII and a part of folium VIc), and the caudal fastigial nuclei (CFN) are crucial in controlling smooth pursuit (33–35). The cerebellum plays a role in fixation stability, impairment of which would lead to increased SWJ (36). The brain structures responsible for normal saccades comprise the paramedian pontine reticular formation (PPRF), the rostral interstitial nucleus of the medial longitudinal fasciculus (riMLF), the OMV, and the CFN (37). The neural network that confers the ability to maintain horizontal eccentric eye positions is distributed over the cerebellum and brainstem comprising the flocculus, the nucleus prepositus hypoglossi, and the adjacent medial vestibular nucleus. Damage to one of these structures causes GEN (38). The affected areas are also wide in patients with SCA3: the degenerative process initially involves the cerebellum and cerebellar nuclei, substantia nigra, and vestibular and oculomotor nuclei, while the thalamus, pons, and medulla oblongata, and cranial nerves from III to XII are later involved (39). A previous study found that GEN was observed in 17% of asymptomatic SCA3 carriers but was absent in non-carrier controls, indicating that GEN may appear preceding other signs in patients with SCA3 (40). Another study also found that several oculomotor alterations could be detected in pre-SCA3 carriers, including SWJ and GEN (29). A study compared the degree of lower extremity ataxia with the degree of oculomotor disorder by using eye tracking tests (ETT) and optokinetic pattern tests (OKP) and found that SCA3 patients showed high ETT and low mean slowest phase velocity in OKP, indicating that SCA3 tends to be characterized by oculomotor disorder preceding extremity ataxia (41). Thus, abnormal eye movement might be a sensitive early biomarker of the impairment of neural networks and brain structures. This may be a reasonable explanation for the predictive role of abnormal eye movements on falls or, more generally, on disease progression in patients with SCA3 detected in the current study.

This is the first study to explore the potential predictive factors of falls in a prospective cohort of patients with SCA3 and to establish a nomogram model to predict falls in SCA3 individual patients. The results showed that pyramidal signs, extrapyramidal symptoms, autonomic dysfunction, and abnormal eye movements are all predictors of falls in SCA3, indicating that SCA3 is not a pure cerebellar disease and that multiple systems are involved in its pathogenesis. However, several limitations should be acknowledged in the current study. The first limitation was the lack of electro-oculography or video-oculography to assess abnormal eye movements objectively. Instead, eye movements were assessed by neurologists who were experienced in movement disorders using accepted bedside techniques described before (12). The second limitation was the lack of external validation of the nomogram model due to the small sample size. Further validation in other patient cohorts is required. Therefore, our findings need validation in an external population before they can be used to counsel patients and their families.

In conclusion, our study revealed that patients with dystonia, hyperreflexia, urinary incontinence, hidrosis, and more types of abnormal eye movement had a more rapid progression to falls in SCA3, which emphasizes the importance of clinical assessment of these characteristics in patients with SCA3. Additionally, our study established the first nomogram to predict falls in patients with SCA3.

## Data Availability Statement

The original contributions presented in the study are included in the article/Supplementary Materials, further inquiries can be directed to the corresponding author/s.

## Ethics Statement

The studies involving human participants were reviewed and approved by the Ethics Committee of West China Hospital of Sichuan University. The patients/participants provided their written informed consent to participate in this study.

## Author Contributions

JL contributed with conception, organization and execution, data collection and statistical analysis, and drafting the manuscript. LZ contributed with execution, data collection, and statistical analysis. BC, YH, KL, XX, and XG contributed with execution and data collection. QW contributed with execution and data collection. RO contributed with conception, organization, execution, and data collection. HS contributed with conception and organization, manuscript review and critique, and responsible for the overall content as the guarantor. All authors contributed to the article and approved the submitted version.

## Conflict of Interest

The authors declare that the research was conducted in the absence of any commercial or financial relationships that could be construed as a potential conflict of interest.

## Supplementary Material

Supplementary Figure 1 Time-dependent receiver operating characteristic curves (t-ROC) of the nomogram prediction. X-axis demonstrates the false positive rate of the model, and Y-axia shows the true positive rate of the model. AUC, areas under the ROC curve; TP, true positive; FP, false positive.

Supplementary Figure 2 Calibration curves of the nomogram prediction.
