# Supplementary figures and images for: Establish a Nomogram to Predict Falls in Spinocerebellar Ataxia Type 3
Source: Front Neurol. 2021 Jan 27;11:602003. doi: 10.3389/fneur.2020.602003 (PMC7873475; doi:10.3389/fneur.2020.602003)

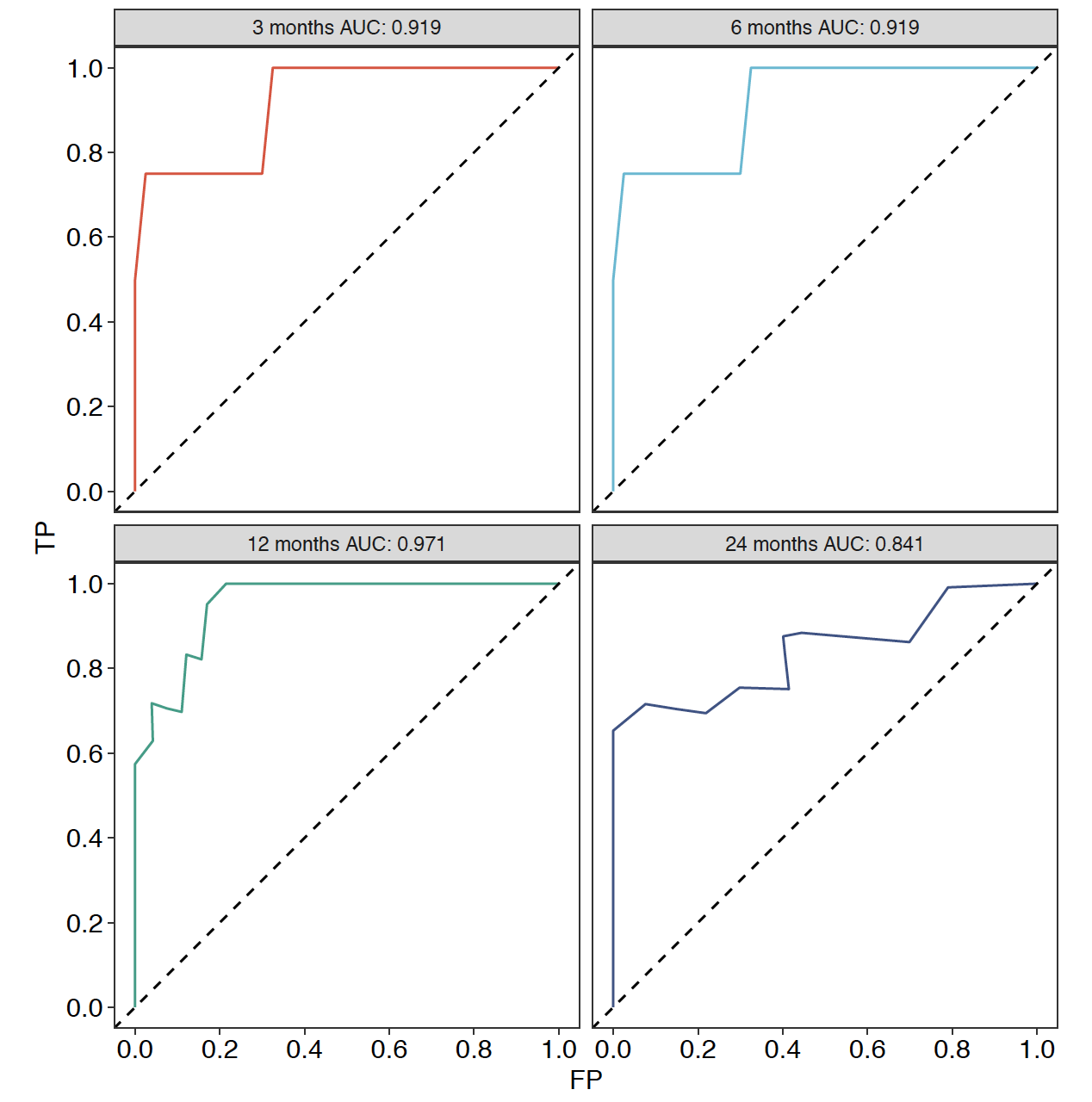

Supplement: Supplementary Figure 1 — Time-dependent receiver operating characteristic curves (t-ROC) of the nomogram prediction. X-axis demonstrates the false positive rate of the model, and Y-axia shows the true positive rate of the model. AUC, areas under the ROC curve; TP, true positive; FP, false positive. [file Image_1.TIFF]

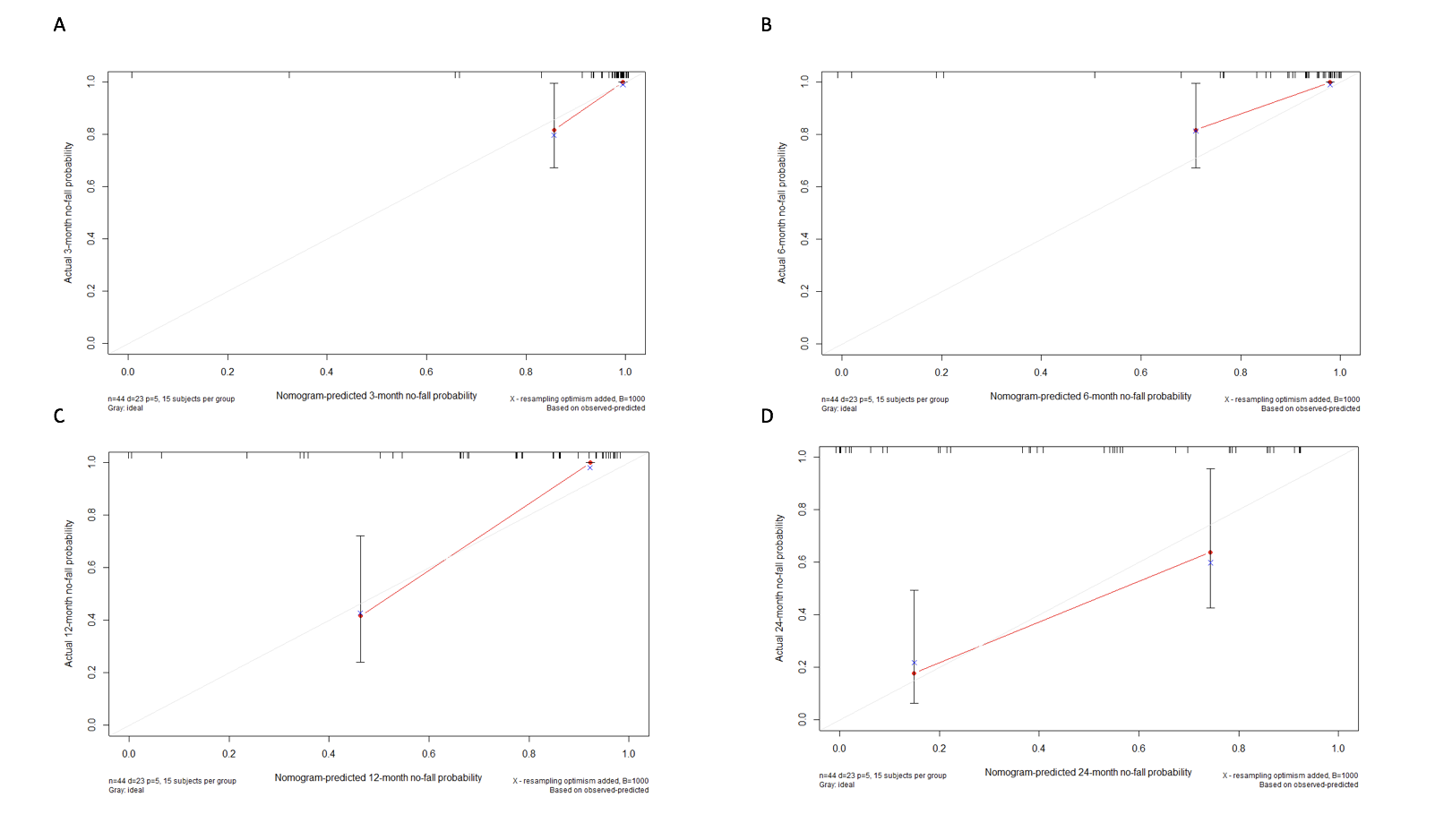

Supplement: Supplementary Figure 2 — Calibration curves of the nomogram prediction. [file Image_2.TIFF]
